# Does oxygen delivery explain interindividual variation in forearm critical impulse?

**DOI:** 10.14814/phy2.12203

**Published:** 2014-11-20

**Authors:** J. Mikhail Kellawan, Robert F. Bentley, Michael F. Bravo, Jackie S. Moynes, Michael E. Tschakovsky

**Affiliations:** 1 Department of Kinesiology, School of Education, University of Wisconsin, Madison, Wisconsin; 2 Human Vascular Control Laboratory, School of Kinesiology and Health Studies, Queen's University, Kingston, Ontario, Canada

**Keywords:** Aerobic capacity, critical power, exercise hyperemia, forearm exercise, pressor response

## Abstract

Within individuals, critical power appears sensitive to manipulations in O_2_ delivery. We asked whether interindividual differences in forearm O_2_ delivery might account for a majority of the interindividual differences in forearm critical force impulse (critical impulse), the force analog of critical power. Ten healthy men (24.6 ± 7.10 years) completed a maximal effort rhythmic handgrip exercise test (1 sec contraction‐2 sec relaxation) for 10 min. The average of contraction impulses over the last 30 sec quantified critical impulse. Forearm brachial artery blood flow (FBF; echo and Doppler ultrasound) and mean arterial pressure (MAP; finger photoplethysmography) were measured continuously. O_2_ delivery (FBF arterial oxygen content (venous blood [hemoglobin] and oxygen saturation from pulse oximetry)) and forearm vascular conductance (FVC; FBF·MAP^−1^) were calculated. There was a wide range in O_2_ delivery (59.98–121.15 O_2_ mL·min^−1^) and critical impulse (381.5–584.8 N) across subjects. During maximal effort exercise, O_2_ delivery increased rapidly, plateauing well before the declining forearm impulse and explained most of the interindividual differences in critical impulse (*r*^2^ = 0.85, *P* < 0.01). Both vasodilation (*r*^2^ = 0.64, *P* < 0.001) and the exercise pressor response (*r*^2^ = 0.33, *P* < 0.001) independently contributed to interindividual differences in FBF. In conclusion, interindividual differences in forearm O_2_ delivery account for most of the interindividual variation in critical impulse. Furthermore, individual differences in pressor response play an important role in determining differences in O_2_ delivery in addition to vasodilation. The mechanistic origins of this vasodilatory and pressor response heterogeneity across individuals remain to be determined.

## Abstract (precis)

We tested the hypothesis that interindividual differences in oxygen delivery would be strongly associated with forearm critical impulse. We found that oxygen delivery explained 85% of the forearm critical impulse differences between individuals and that differences in the pressor response between individuals in addition to differences in vasodilation accounted for differences in oxygen delivery. The mechanistic origins of this interindividual variation remain to be determined.

## Introduction

Critical power is the maximal power output at which a metabolic steady state characterized by a plateau in 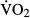, stable intracellular levels of Pi, PCr, ADP, ATP, H^+^, and stable blood lactate is reached (Poole et al. 1988; Jones et al. 2008, 2010). Exercising above critical power is limited by factors determining the fixed supracritical power work capacity, termed W′, and results in progressively increasing 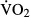, intracellular [Pi] and blood lactate until exhaustion at which point 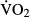 is at maximum (Jones et al. 2008).

Traditionally, critical power is identified within an individual by determining the asymptote of their power–duration relationship from 3 to 4 constant load tests to exhaustion. Recently, critical power obtained in this manner has been found to be sensitive to manipulations in O_2_ delivery. For example, Vanhatalo et al. (2010) found that the power–duration curve asymptote was shifted upwards when exercise was performed while breathing hyperoxic air, while Dekerle et al. (2012) demonstrated that it was shifted downwards when exercise was performed while breathing hypoxic air. These findings support the contention that critical power represents the maximal power output at which aerobic ATP production can meet ATP demand without requiring additional substrate level phosphorylation (Dekerle et al. 2012), and that this aerobic ATP production rate is sensitive to manipulation of O_2_ delivery to the exercising muscle within an individual.

Critical power can also be determined using a single bout, maximal effort exercise protocol, validated for upright cycling (Burnley et al. 2006), knee extension (Burnley 2009) and most recently forearm handgrip exercise (Kellawan and Tschakovsky 2014). In this type of critical power identification test, subjects are required to produce maximal effort exercise that results in an exponential decay in power output to a plateau which represents critical power. It has been demonstrated that this method provides valid estimates of critical power (Burnley et al. 2006; Vanhatalo et al. 2007; Kellawan and Tschakovsky 2014). As critical power represents the upper limit of sustainable work rates, interindividual differences in critical power reflect interindividual differences in the range of tolerable exercise work rates. In this context, identifying factors that contribute to interindividual differences in critical power has important implications for understanding exercise (in)tolerance at the level of the individual.

Studies employing within‐subject manipulation of O_2_ delivery, while providing evidence in support of an O_2_ delivery sensitivity of critical power (Vanhatalo et al. 2010; Dekerle et al. 2012), cannot address the question of whether interindividual differences in O_2_ delivery contribute to interindividual differences in critical power. Instead, we reasoned that if the answer to this question was “yes”, then under the conditions of a single–bout maximal effort critical power test in which 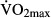 is rapidly attained (Burnley et al. 2006) we would have to observe two things. First, there would have to be a strong association between interindividual variation in O_2_ delivery and critical power. Second, to rule out that such an association is only because the O_2_ demand of a stable plateau in contraction impulse is determining the O_2_ delivery response, O_2_ delivery would have to achieve a stable plateau well before power output. Should these criteria be satisfied, then the partitioning of exercising muscle vasodilatory versus pressor response contribution to these differences in O_2_ delivery between individuals would provide additional insight into the relative importance of each in determining an individual's O_2_ delivery response.

Therefore, the objectives of this study were to identify to what extent interindividual differences in O_2_ delivery are associated with interindividual differences in critical power obtained from a single–bout maximal effort exercise test, whether O_2_ delivery stabilizes before power output, and finally to what extent any such interindividual differences in O_2_ delivery are due to differences in vasodilatory versus pressor responses.

As a first step, we pursued this objective at the level of isolated small muscle mass exercise, where determinants of exercising muscle O_2_ delivery are dependent on local vasoregulatory and systemic pressor responses, using our recently validated single–bout maximal effort forearm isometric handgrip exercise test (Kellawan and Tschakovsky 2014). In this type of exercise the contraction impulse represents the force analog of power output, and forearm critical force impulse (critical impulse) represents the force analog of critical power. We hypothesized that interindividual differences in O_2_ delivery would account for a majority of the interindividual variation in critical impulse, and a plateau in O_2_ delivery would clearly precede that for contraction impulse. Further to this we hypothesized that interindividual differences in vasodilatory response would account for the majority of interindividual variation in O_2_ delivery.

## Materials and Methods

### Subjects

Ten healthy men (age 24.6 ± 7.10 years) volunteered to participate in the study. The participants were recreationally active (268.15 ± 25.68 metabolic equivalents/week; most of the activity was from participation in team sports that involved the whole body and running, but did not participate in forearm exercise training). Participant's characteristics are outlined in Table 1. After receiving a complete verbal and written description of the experimental protocol and potential risks, each subject provided signed consent to the experimental procedures that were approved by the Queen's University Health Sciences Research Ethics Board (QUHSREB) in accordance with the terms of the Declaration of Helsinki on research ethics.

**Table 1.** Subject characteristics.

Subject (#)	Age (years)	Height (cm)	Weight (kg)	Forearm volume (mL)	Forearm Girth (cm)	[Hb] (g·dL^−1^)	S_a_O_2_ (%)	C_a_O_2_ (mL O_2_·dL^−1^)
1	21	166	58	780	28.10	16	100	21.74
2	23	181	76	1054	27.40	14.4	98	19.25
3	44	188	84.5	1160	28.50	14.6	100	19.86
4	22	176	74	1232	23	13.7	99	18.47
5	22	174	71.5	991	24	15.1	100	20.53
6	21	178	81	1354	28.3	15.1	97	20.02
7	25	178	91.5	1184	28.5	16.9	100	22.71
8	20	179	77	1114	27.4	15.9	97	21.00
9	27	178.5	87	1374	29	15.4	98	20.52
10	21	192	89.5	1174	27	14.8	100	20.52
Mean	24.7 ± 7.1	179.1 ± 7.1	79 ± 10	1141.7 ± 174.0	27.1 ± 2.0	15.2 ± 0.9	98.9 ± 1.2	20.5 ± 1.2

Individual values. Last row includes mean ± SD.

### Experimental design

#### Maximal effort forearm critical force impulse test

All experimental sessions were conducted in a temperature‐controlled laboratory (20–22°C) after a minimum 2 h fast and 12 h without exercise and caffeine. The subjects lay supine with the experimental arm (left) extended 90° at heart level. After 2 min of quiet rest, subjects completed 10 min of rhythmic, maximal effort isometric handgrip contractions (1 sec contraction to 2 sec relaxation). This type of exercise results in a roughly exponential decay in contraction impulse (the area under the contraction force tracing) to an eventual plateau, the magnitude of which represents critical impulse (Kellawan and Tschakovsky 2014). Subjects received continuous, strong verbal encouragement, and coaching of contraction performance throughout the test to ensure that they gave a maximal effort during each contraction and to maintain the work‐to‐rest duty cycle.

### Measurements

#### Mean arterial blood pressure (MAP) and heart rate (HR)

Beat by beat MAP (mmHg) was measured by finger photoplethysmography (Finometer MIDI, Finapres Medical Systems BV, Amsterdam, the Netherlands) on the middle finger of the control arm resting at heart level during all trials. HR was acquired using a three‐lead ECG (Meditrace 535; Tyco HealthCare Group, Pointe Claire, Quebec, Canada).

#### Forearm blood flow (FBF) and vascular conductance (FVC)

Mean blood velocity (MBV, cm·sec^−1^) was measured in the brachial artery with a 4‐MHz pulsed Doppler probe (model 500V TCD; Multigon Industries, Mt. Vernon, NY) secured to the skin over the brachial artery. To maximize the between‐subject comparability of our forearm blood flow measures, we have calibrated our Doppler ultrasound probe with known flow velocities, and we measure the angle of the brachial artery relative to the skin surface (i.e., relative to the probe insonation angle). This allows us to correct for differences in insonation angle that may exist between subjects. This has been previously described in detail (Tschakovsky et al. 2011). Brachial artery diameter was measured just proximal to the Doppler probe placement (10‐MHz linear echo Doppler ultrasound probe operating in B‐mode, Vingmed System Five, GE Medical, Mississauga, Ontario, Canada). FBF (mL·min^−1^) was calculated from MBV and artery diameter (FBF = MBV·60 sec·min^−1^·*π*·(brachial artery diameter·2^−1^)^2^), while forearm vascular conductance (FVC;mL·min^−1^·mmHg^−1^) was calculated from FBF and MAP (FBF·MAP^−1^).

#### Hemoglobin concentration ([Hb]), % saturation (S_a_O_2_), arterial oxygen content (C_a_O_2_), and oxygen delivery

A venous blood sample was taken from the anticubital vein and analyzed for Hb content by an automated blood gas analyzer (Stat Profile M, Nova Biomedical, Waltham, MA). Arterial oxygen saturation (S_a_O_2_) was measured from the index finger of the control arm using pulse oximetry (Nellcor, the Netherlands). Oxygen content of arterial blood (C_a_O_2_) was calculated: 1.34 mL O_2_·g Hb^−1^·g Hb·mL of blood^−1^·% S_a_O_2_ + 0.003 mL O_2_·mL of blood^−1^. O_2_ delivery (mL O_2_·min^−1^) was calculated by multiplying C_a_O_2_ and FBF.

### Data acquisition and analysis

MAP, HR, and MBV data were collected at 200 Hz with a data acquisition system (Powerlab, ADInstruments, Colorado Springs, CO) and recording computer. Brachial artery images were recorded in Digital Imaging and Communications in Medicine (DICOM) format for offline measurement of brachial artery diameter using custom automated edge‐detection software (Woodman et al. 2001). Baseline and hyperemic values of MAP, HR, FBF, and calculated FVC were obtained on a beat‐by‐beat basis. These values were averaged over 3 sec (contraction/relaxation duty cycle duration) time bins. Brachial artery images were recorded continuously and diameter measurements performed on clear images during relaxation selected at 30 sec intervals. Each individual's diameter data were then curve fit using an exponential rise to maximum nonlinear regression. One, two, or three component models were used depending on which model fit of an individual's data resulted in the residuals being equally distributed and of the smallest magnitude possible using custom software as previously described in Pyke and Tschakovsky (2007). O_2_ delivery area under the curve calculations was derived from every individual's 3 sec avg profile during exercise.

Contraction impulse for each duty cycle was calculated as the area under the force displacement curve for each contraction. The critical impulse was calculated from the average of the contraction impulses generated during the last 30 sec (~10 contractions) of the exercise test. To compare time to plateau of contraction impulse and O_2_ delivery responses, a low‐pass filter was applied to each subject's contraction impulse and O_2_ delivery profiles as outlined by Ferreira et al. (2005). The Δcontraction impulse and ΔO_2_ delivery values were then averaged over 3 sec time bins and normalized for each subject from 0%, corresponding to rest for O_2_ delivery and the first contraction for contraction impulse to 100% reflecting the stable plateau values (values averaged over the last 30 sec of the test). The average of five 3 sec time bins every 30 sec was used for statistical analysis to identify when a stable plateau was reached.

### Statistical analysis

Two‐way repeated measures ANOVA was performed on the % of stable plateau in contraction impulse and O_2_ delivery responses in order to identify time points where these variables differed as well as when within a variable the response was no longer different from the stable plateau.

Pearson moment correlation and forward stepwise linear regression was used to determine how much interindividual variation in critical impulse, O_2_ delivery and FBF was accounted for by interindividual differences in variables of interest. For critical impulse the variables included in the forward stepwise regression were forearm characteristics (volume and girth), maximal voluntary contraction and O_2_ delivery. For O_2_ delivery these variables were FBF and C_a_O_2_. For ΔFBF these variables were ΔFVC and ΔMAP. All analyses were performed using Sigmaplot 11, Systat Software, Chicago, IL. Significance was set a prori at *P* ≤ 0.05.

## Results

### Baseline characteristics

Subject characteristics can be viewed in Table 1.

### Interindividual variation in critical impulse: which variables account for this?

**Figure 1.**
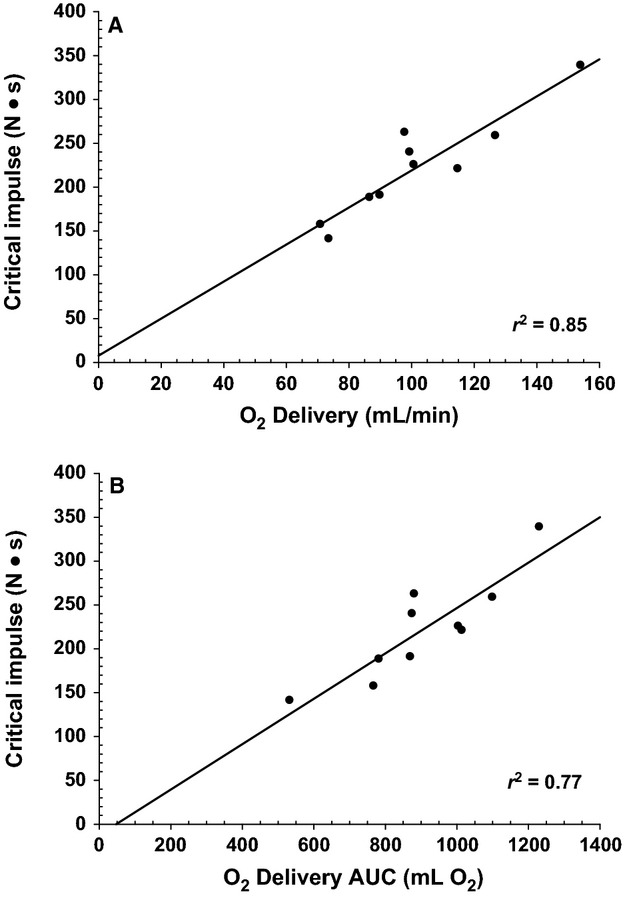
Relationship between O_2_ delivery and forearm critical impulse in healthy male subjects. Panel A: Critical impulse versus O_2_ delivery as quantified by the last 30 sec of the maximal effort test (*r* = 0.92, *r*^2^ = 0.85, *P* < 0.01). Panel B: critical impulse versus O_2_ delivery as quantified by the total amount of O_2_ delivered (area under the curve; AUC) (*r* = 0.88, *r*^2^ = 0.76, *P* < 0.01).

**Figure 2.**
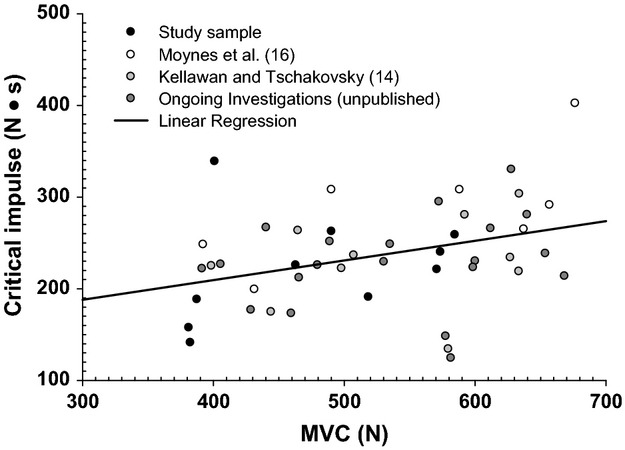
Relationship between maximal voluntary contraction (MVC) and forearm critical impulse in healthy male subjects. Data pooled over multiple studies from our laboratory. Individual study data is identified in the legend. Regression line: Critical Impulse = 123.404 + (0.215 MVC), *r*^2^ = 0.125, *P* = 0.015.

O_2_ delivery during the maximal effort test varied substantially between subjects (O_2_ delivery AUC range 533–1231 mL·O_2_, plateau in O_2_ delivery 73.59–154.10 mL O_2_·min^−1^) as did critical impulse (381.5–584.8 N). Forward stepwise regression of O_2_ delivery plateau and forearm characteristics against critical impulse identified O_2_ delivery as a strong predictor of critical impulse (O_2_ delivery *r* = 0.92, *r*^2^ = 0.85, *P* < 0.001), whereas forearm characteristics fell out of the model as they had no relationship with critical impulse (MVC *P* = 0.969, forearm volume *P* = 0.639, forearm girth *P* = 0.381) The O_2_ delivery AUC was also a strong predictor, explaining 77% of the variance in critical impulse when regressed separately (O_2_ delivery AUC *r* = 0.88, *r*^2^ = 0.77, *P* < 0.01) (Fig. 1A and B). In contrast, the ΔO_2_ delivery from baseline accounted for only 66% of the variance in critical impulse. Furthermore, while forearm volume was significantly correlated with MVC (*r*^2^ = 0.42, *P* = 0.04) forearm girth was not (*r*^2^ = 0.06, *P* = 0.48). However, addition of subjects from previously published studies (Moynes et al. 2013; Kellawan and Tschakovsky 2014) as well as unpublished data from ongoing investigations increasing the total n to 47 identified a weak, albeit statistically significant,, relationship between critical impulse and MVC (Fig. 2; *r*^2^ = 0.125, *P* = 0.015).

### Which of O_2_ delivery and critical impulse achieves a stable plateau first?

**Figure 3.**
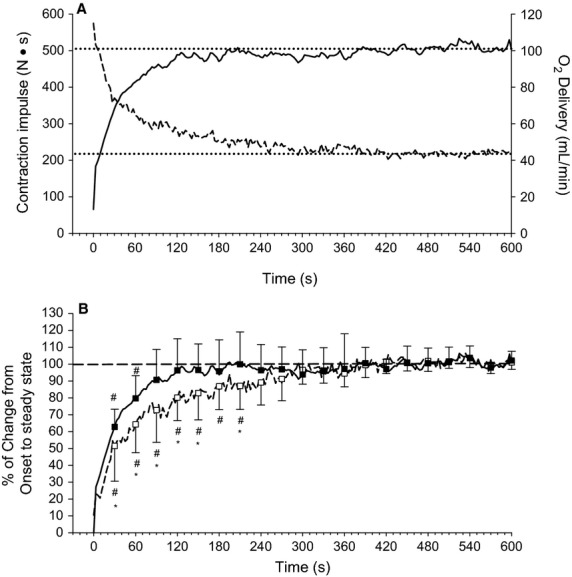
Change in O_2_ delivery and contraction impulse during maximal effort exercise. Dotted lines identify stable plateau. Panel A: absolute changes. Dashed line is contraction impulse. Solid line is O_2_ delivery. Panel B: expressed as % of the change from baseline to steady state for O_2_ delivery and from the first maximal effort contraction to steady state for contraction impulse. Dashed line and open squares is contraction impulse. Solid line and closed squares is O_2_ delivery. ^#^Significantly different from steady state within a variable. *Significantly different from O_2_ delivery at a given time point. All *P* < 0.05.

Figure 3 illustrates the time course of changes in O_2_ delivery and contraction impulse over the course of a 10 min, maximal effort handgrip exercise test. O_2_ delivery increased to a stable plateau, whereas maximal effort contraction impulse declined to a stable plateau (Fig. 3A). The magnitude of change from onset to plateau is expressed as % of change from onset to plateau in Fig. 3B in order to facilitate comparison of the time to plateau in O_2_ delivery versus contraction impulse. The increase in O_2_ delivery to stable plateau is no longer statistically significantly different from stable plateau by 90 sec (*P* = 0.48), whereas the contraction impulse remains statistically significantly below steady state and lower than the change in O_2_ delivery up to 210 sec (*P* = 0.03 and *P* = 0.02, respectively, at 210 sec), confirming that O_2_ delivery stabilizes well before contraction impulse during this type of exercise.

### How much interindividual variation in O_2_ delivery is accounted for by interindividual differences in FBF and arterial oxygen content (C_a_O_2_)?

Forward stepwise linear regression analysis identified that both FBF and C_a_O_2_ contributed independently to interindividual differences in steady‐state O_2_ delivery, but FBF was by far the stronger determinant accounting for ~94% of interindividual differences in our study sample (Table 2).

**Table 2.** Forward stepwise linear regression quantifying the independent contribution of forearm blood flow (FBF) and arterial oxygen content (C_a_O_2_) to interindividual differences in O_2_ delivery.

Dependent variable	Independent variable	Δ *r*^2^ by addition to model	*r* ^2^	*P* value
O_2_ delivery	FBF	0.944	0.944	<0.001
	C_a_O_2_	0.0515	0.996	<0.001
Total regression			0.996	<0.001

### How much interindividual variation in FBF is accounted for by FVC and MAP?

**Figure 4.**
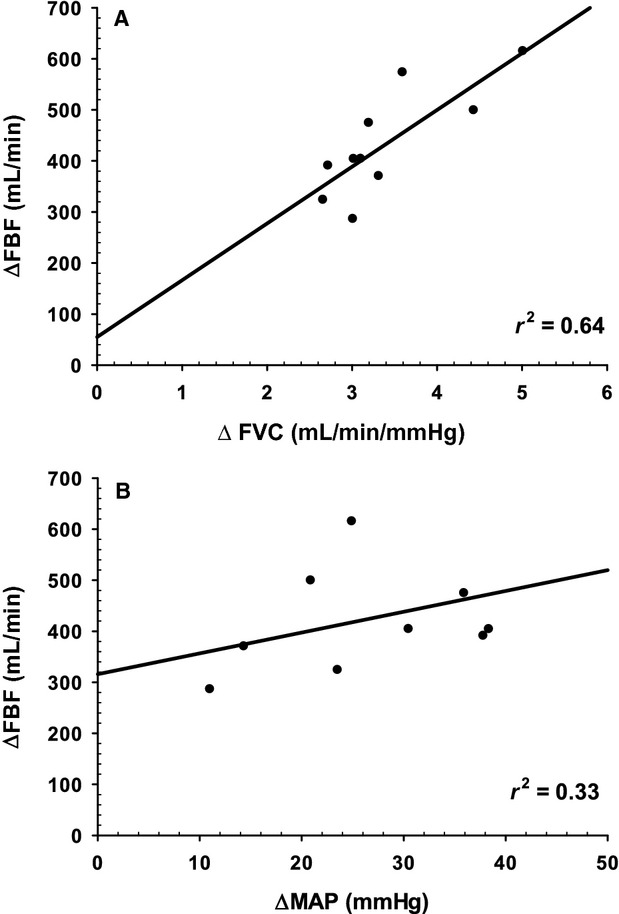
ΔFVC and ΔMAP from baseline as predictors of ΔFBF. Panel A: ΔFBF versus ΔFVC as quantified by the difference between rest and the last 30 sec of the maximal effort test (*r*^2^ = 0.64, *P* < 0.001). Panel B: ΔFBF versus ΔMAP as quantified by the difference between rest and the last 30 sec of the maximal effort test (*r*^2^ = 0.33, *P* < 0.001).

When assessed separately with simple linear regression, correlation of ΔFBF with ΔFVC from baseline was statistically significant (*r* = 0.80, *r*^2^ = 0.64, *P* = 0.006) but not with ΔMAP (*r* = 0.49, *r*^2^ = 0.235, *P* = 0.15). However, forward stepwise linear regression analysis revealed that the addition of ΔMAP to ΔFVC substantially improved the model's ability to account for interindividual variation in FBF (Table 3 and Fig. 4).

**Table 3.** Forward stepwise linear regression quantifying the independent contribution of forearm vascular conductance (FVC) and mean arterial pressure (MAP) to interindividual differences in FBF. Δ – change from baseline. AUC – area under the curve of the total response. test.

Dependent variable	Independent variable	Δ *r*^2^ by addition to model	*r* ^2^	*P* value
Δ FBF	Δ FVC	0.63	0.63	<0.001
	Δ MAP	0.34	0.97	<0.001
Total regression			0.97	<0.001
FBF AUC	Δ FVC AUC	0.51	0.51	<0.001
	Δ MAP AUC	0.39	0.89	0.002
Total regression			0.89	<0.001

## Discussion

In support of the construct that critical power represents the highest power output that can be sustained via oxidative phosphorylation such that 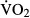, PCr and pH stabilize, investigators have provided indirect evidence for its oxygen dependence (Vanhatalo et al. 2010; Dekerle et al. 2012). These experiments involved the within‐subject manipulation of arterial oxygen content via hypoxic and hyperoxic inspired gas, inferring altered O_2_ delivery to exercising muscle. They demonstrated that critical power was increased with hyperoxia and decreased with hypoxia.

However, these studies were not intended to answer the question of whether and to what degree interindividual differences in O_2_ delivery can account for interindividual variation in critical power. By its very nature, this question cannot be answered by manipulation of O_2_ delivery within subjects. Instead, evidence has to come from the strength of association of interindividual variation in O_2_ delivery and critical power and the relative timing of a stable plateau in O_2_ delivery versus critical power. Therefore, we tested the hypothesis that interindividual differences in O_2_ delivery would account for a majority of the interindividual variation in critical impulse, the force analog of critical power, and that a plateau in O_2_ delivery would precede a plateau in contraction impulse. Further to this, we tested the hypothesis that interindividual differences in vasodilatory response would account for the majority of interindividual variation in O_2_ delivery.

The novel findings of this investigation were as follows. First, interindividual differences in O_2_ delivery, which were predominantly due to FBF with only a small contribution from C_a_O_2_, explained 85% of the interindividual variation in critical impulse. Second, the increase in O_2_ delivery reached a plateau well before the decline in forearm contraction impulse. This rules out the possibility that the strength of association is simply because O_2_ delivery is adjusting to meet a set metabolic demand but supports the possibility that the established O_2_ delivery determines the sustainable metabolic demand. Taken together, these two findings are consistent with the hypothesis that inherent interindividual differences in O_2_ delivery is an important determinant of interindividual differences in critical impulse. Third, vasodilatory response differences between individuals independently accounted for a majority of the interindividual differences in FBF during maximal effort forearm exercise. However, pressor response differences between individuals also accounted for a significant amount of interindividual FBF variation. These findings are the first to draw attention to the potential importance of interindividual differences in the maximal effort exercise pressor response as a determinant of interindividual differences in FBF. Finally, forearm critical force within the study sample was unrelated to MVC, forearm girth, or forearm volume. However, when we included data from published studies (Moynes et al. 2013; Kellawan and Tschakovsky 2014) and other ongoing unpublished work, there was a weak, albeit statistically significant, relationship between MVC and critical impulse.

### O_2_ delivery explains interindividual variation in critical impulse

The absolute O_2_ delivery to the forearm was the strongest predictor of critical impulse (Table 2 and Fig. 1A and B, *r*^2^ = 0.85) and was considerably stronger than Δ O_2_ delivery from rest (*r*^2^ = 0.66). This suggests that absolute O_2_ delivery is the more relevant O_2_ delivery for critical impulse. The most plausible explanation is that O_2_ delivery at baseline is an important part of the O_2_ available to tap into by the working muscle when its demand for O_2_ increases.

The strong association between O_2_ delivery and the stable contraction impulse plateau (critical impulse) could be due to one of two reasons. Either the stabilized contractile work rate representing ATP demand was determining the stabilized O_2_ delivery magnitude (O_2_ demand determining O_2_ supply), or the stabilized O_2_ delivery was constraining the contractile work rate that could be maintained (O_2_ supply determining O_2_ demand). By continuously measuring both contraction impulse and O_2_ delivery throughout the maximal effort test, we were able to assess which of these two variables stabilized first. As matching of ATP demand with aerobic ATP supply is a required condition of sustainable exercise characterized by stable 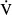
O_2_, PCr, Pi, ADP, and pH, the variable that plateaus first would dictate or constrain the level at which the other variable plateaus.

We therefore quantified the ΔO_2_ delivery and the Δ contraction impulse over the course of the maximal effort test as a percent of their stable plateau (i.e., the plateau of these variables represented 100% of their response, and so changes from rest could be quantified as a % of the plateau (Fig. 3A and B). Statistical analysis could then determine when a variable was no longer different from its stable plateau. This analysis identified that O_2_ delivery reached a stable plateau well before contraction impulse. Stabilization of O_2_ supply prior to demand could not occur if demand was determining supply, effectively ruling out this scenario. Instead, this temporal order of stabilization is consistent with the hypothesis that interindividual variation in O_2_ delivery is an important determinant of interindividual variation in critical impulse.

Further support strengthening this interpretation comes from what is known about the 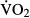 response to maximal effort exercise or fixed exercise above 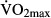. At present, data on the temporal profile of changes in power output and 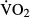 relative to each other during maximal effort exercise have not been documented. However, it has been indicated by Jones et al. (2010) that in their studies employing the 3‐min all‐out cycle ergometer exercise test, all subjects have reached 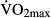 by ~60 sec of exercise, well before the stabilization of power output near the end of such a test. The same time to peak has also been observed with constant load exercise that is 20 to 30% above 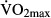 (Hughson et al. 2000; Adami et al. 2011). These are power outputs that are below those occurring during the first minute of a 3‐min all‐out cycling test. The point being that aerobic ATP supply appears to plateau before ATP demand in maximal effort exercise.

While the forearm exercise protocol in our study involved maximal effort contractions, the fixed 2 sec duration of relaxation between contractions meant that the plateau in contraction impulse required substantially more than 3 min, indicating that the rate of depletion of W′ was slower compared to the maximal effort cycling exercise test (Burnley et al. 2006; Vanhatalo et al. 2007). Nevertheless, the metabolic demand of maximal effort contractions during the time prior to achievement of the contraction impulse plateau would have been well above 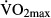. Thus, while we did not measure forearm 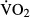 in our study, it is most likely the case that 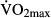 was also reached with a similar time course to the above‐mentioned supramaximal exercise protocols.

In contrast, contraction impulse continued to decline well past this time point, being greater than the critical impulse plateau until 210 sec of exercise (Fig. 3), whereas O_2_ delivery was no longer different from plateau by 90 sec of exercise (Fig. 3). When taken together with observations from other studies that muscle deoxygenation plateaus rapidly during supramaximal exercise (Adami et al. 2011) stability not only in O_2_ delivery but also in 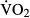 would appear to occur well before the decline in contractile work stabilized in our study. This means that the stable contractile work achieved by the end of the maximal effort test would also represent a stable metabolic demand. This further supports the plausibility of the hypothesis that interindividual differences in O_2_ delivery is actually a primary determinant of interindividual differences in critical impulse.

We therefore propose the following sequence of events during the maximal effort critical impulse test, based on what is known about W′ and critical power and the findings in our study. At the onset of repeated maximal effort contractions, motor unit recruitment to produce force initiates an increased ATP demand to levels well above the maximal rate of aerobic ATP supply. All three energy systems contribute to the provision of ATP, with the difference between aerobic ATP supply (which is increasing rapidly) and demand being made up by PCr hydrolysis and anaerobic glycolysis. However, force production quickly begins to decline even as O_2_ delivery and aerobic ATP supply is rapidly increasing. It is not decreased motor drive as exercise continues that is responsible for the decrease in force that is observed (Bigland‐Ritchie et al. 1983). Rather, accumulation of intracellular substances interfering with excitation contraction coupling ([P_i_] from PCr hydrolysis, [ADP] from imbalance between ATP hydrolysis and ATP regeneration, [H^+^] associated with anaerobic glycolysis) (Fitts 2008; Westerblad et al. 1991; Nelson and Fitts 2014) would be major contributors to the decrease in force.

While it is acknowledged that changes in efficiency (ATP cost per unit of power output) can occur in heavy to severe exercise domains (Poole et al. 1988, 1991, 1994; Poole 1994) and therefore contraction impulse is not perfectly representative of ATP demand, the magnitude of contraction impulse decline is so substantial that overall ATP demand is likely decreasing considerably despite any potential reductions in efficiency. Therefore, in addition to fatigue this decrease would reflect force conforming to the decline in the rate at which ATP can be supplied above maximal aerobic ATP supply under the rapidly changing intracellular environment. Thus, both fatigue and reductions in ATP supply rate would be contributing to declining contraction impulse until the fixed capacity for work above critical impulse (W′) is completely depleted.

Part of this fixed ATP supply in excess of critical power comes from PCr stores, and part of it would come from anaerobic glycolytic ATP production (with small initial contribution from O_2_ stores as well). However, the PCr stores are fixed and depletion means they must eventually stabilize when contraction impulse plateaus at critical impulse, meaning that they have ceased to contribute to ATP supply. In contrast, although the rate of anaerobic glycolysis is likely declining during the course of the maximal effort exercise it must still be contributing to ATP demand as critical impulse is reached, as blood lactate and pH are known to stabilize at levels considerably different from rest (Poole et al. 1988) under conditions where mechanisms responsible for lactate and H^+^ removal from the blood remain active (Brooks 1986, 1991; Stanley et al. 1986). Since by definition exercise at critical impulse would mean there is stability of pH and blood lactate, then this rate of anaerobic glycolysis could represent the maximal anaerobic glycolytic flux that can still be balanced by these lactate and H^+^ removal mechanisms. At this point ATP metabolic demand (contraction impulse) that can be sustained must conform to the maximal rate of aerobic ATP production plus the sustainable anaerobic glycolytic ATP production. The maximal rate of aerobic ATP production would be dependent on the interaction of convective O_2_ delivery, diffusive conductance of skeletal muscle, and mitochondrial content (Tschakovsky and Pyke 2008), and therefore the observed individual differences in critical impulse that were established well after stability in O_2_ delivery would be dependent in part on O_2_ delivery differences.

The implications of our findings are that differences between individual's ability to supply muscle with O_2_ during maximal effort exercise may be partly responsible for interindividual differences in critical impulse. According to the power–duration relationship, differences in critical impulse between individuals represent differences in the range of exercise intensities that would be considered sustainable (Jones et al. 2008, 2010), translating to differences in exercise tolerance.

### Vasoregulatory versus pressor mechanism contributions to forearm blood flow

FBF accounted for virtually all of the interindividual difference in O_2_ delivery. Thus in this group of young healthy subjects, oxygen carrying capacity plays only a very minor role in determining individual differences in O_2_ delivery. This may reflect the relatively small range of CaO_2_ across individuals in our sample. It would be expected that as this range increased across a sample, the potential for CaO_2_ to explain more of the interindividual differences in O_2_ delivery with maximal effort exercise would increase.

To quantify the magnitude of the vasodilatory and pressor response contribution to the ΔFBF response in the critical impulse test, we examined the ΔFVC and Δ MAP from baseline, as these represent the actual compensation that has to occur when metabolic demand for O_2_ increases. Our findings indicate that it was not only the magnitude of vasodilation that determined the FBF response. Rather, interindividual differences in the magnitude of the exercise pressor response also made an important contribution (Table 3).

While changes in MAP have been shown to contribute significantly to exercising limb perfusion recovery in response to experimentally evoked ischemia (Rowell et al. 1991), to our knowledge there has been virtually no attention paid to the potential importance of the magnitude of the pressor response during exercise in determining the magnitude of exercising limb blood flow. For instance, the seminal work of Andersen and Saltin (Andersen and Saltin 1985) which identified that maximal muscle blood flow was far greater than previously thought has since been discussed from the perspective of skeletal muscle vasodilatory capacity and its implications for blood pressure regulation (Richardson et al. 2000). However, observations of vascular conductance plateauing at work rates below peak during incremental small muscle mass exercise while blood flow continues to increase (Mortensen et al. 2005; Saunders et al. 2005) suggests an important role for pressor mechanisms in determining the magnitude of the muscle blood flow response. In the case of Mortensen et al. (2005), over 30% of the maximal single–leg knee extension exercise blood flow response was due to the pressor response.

Our forward stepwise regression analysis identified how much vasodilation versus the pressor response contributed to interindividual differences in maximal effort exercise FBF. Sixty‐four percent of the interindividual variation in FBF was due to vasodilation, whereas 33% was attributable to the pressor response. The findings point to the importance of considering individual differences in pressor response magnitude in addition to vasodilation as a determinant of maximal effort small muscle mass exercise blood flow.

The magnitude of elevation in MAP during maximal or near maximal effort fatiguing exercise is thought to be exercise intensity but not muscle mass dependent (Williams 1991). Contributors to the exercise pressor response include a resetting of the baroreflex which is mediated by central command and muscle mechano‐ and metabo‐reflexes, as well as independent effects of the muscle metabo‐reflex (Gallagher et al. 2006). Therefore, it is possible that interindividual differences in the pressor response in our study may be explained by these mechanisms. This awaits further investigation.

The maximal increase in vascular conductance during a maximal effort test could be constrained by the structural limitation imposed by the number and size of resistance vessels, and/or a functional vasodilatory capacity which would be dependent not only on vasodilatory mechanisms but also on the degree of sympathetic vasoconstrictor activation and its interaction with local sympatholytic factors. Understanding the factors that determine the magnitude of exercise pressor and vasodilatory responses to exercise and the mechanisms that link them to the attempt to match O_2_ delivery to metabolic demand would therefore seem important in the context of understanding exercise intolerance differences between individuals. These may predispose to differences in activity levels and subsequent sedentary related disease development.

### Peak force generation and critical impulse

As in our previous work (Kellawan and Tschakovsky 2014) interindividual differences in MVC (range 381.5–584.8N) were poorly correlated with critical impulse. The same was true for our indirect measures of muscle volume (forearm volume, range 780–1374 mL) and muscle cross‐sectional area (forearm girth, 23–29 cm). Interestingly, while forearm volume was significantly correlated with MVC, forearm girth was not. However, when we pooled data from our laboratory that were previously published (Moynes et al. 2013; Kellawan and Tschakovsky 2014) as well as ongoing findings not yet published with a resulting *n* of 47, MVC accounted for a statistically significant 12.5% of the variation in critical impulse (Fig. 2). These data are consistent with the findings of Saugen et al. (1997) and Kent‐Braun et al. (1993) who demonstrated that reductions in PCr and pH during exercise at the same % MVC can be drastically different between individuals. This suggests that mechanisms relating to an individual's maximal force generating capacity are most likely minor determinants of exercising aerobic metabolic power.

### Potential limitations

We were unable to measure a number of skeletal muscle specific characteristics that could potentially contribute to interindividual differences in critical impulse such as fiber type, buffering capacity, and mitochondrial content. It is quite possible that some or all of these would covary with O_2_ delivery as well as have independent association with critical impulse. However, there would have to be a substantial covariation before the independent contribution of O_2_ delivery would become physiologically insignificant. Finally, had these other characteristics been of a wide enough range and exerted significant independent influence on critical impulse, the variation in critical impulse that could be accounted for by O_2_ delivery as observed in this study would have been lowered considerably.

Another potential issue is that, because interindividual differences in O_2_ delivery were almost completely due to differences in FBF, there is the potential for an effect of blood flow‐dependent removal of fatiguing substances from the skeletal muscle cytosol (Barclay 1986) on critical impulse. However, careful consideration of the determinants of W′ and critical impulse discussed previously would suggest that the magnitude of such a contribution independent of O_2_ delivery support of aerobic metabolism is at best minor. For example, it remains unknown whether blood flow has an impact on intracellular levels of Pi via a “washout” effect. However, since anaerobic glycolysis must still be contributing to ATP demand as critical impulse is reached, it is conceivable that increased blood flow dependent “washout” of H^+^ could allow for a greater rate of anaerobic glycolysis to occur at the lowest tolerable cytosolic pH and thereby increase total ATP production and support a greater critical impulse. However, it seems highly unlikely that the potential impact on ATP production would be of sufficient magnitude to result in a detectable impact on differences in critical impulse between individuals. Nevertheless, it must be acknowledged as a potential mechanism.

## Conclusions

In summary, we have demonstrated for the first time that O_2_ delivery to the exercising forearm during a maximal effort critical impulse test varies considerably across individuals and is a strong predictor of an individual's critical impulse. The additional finding that the increase in O_2_ delivery stabilizes well before contraction impulse rules out the possibility that the association between O_2_ delivery and critical impulse is simply a result of O_2_ delivery adjusting to meet an established metabolic demand. Instead, it is consistent with the hypothesis that these differences in O_2_ delivery between individuals causally contribute to the interindividual variation in critical impulse. This hypothesis remains to be tested. Of considerable interest was the finding that both vasodilatory and exercise pressor responses contributed to interindividual differences in FBF and subsequently O_2_ delivery. This implies that small muscle mass exercise tolerance differences between individuals may be explained not only by differences in the magnitude of vasodilation response, but also by the pressor response. Understanding the underlying mechanistic basis for inherent interindividual differences in cardiovascular support of exercising muscle is an important next step in identifying contributors to exercise intolerance.

## Acknowledgments

We would like to acknowledge the subjects for their time and effort. We would also like to acknowledge Rebecca Shantz for her technical assistance in data collection.

## Conflict of Interest

None declared.
